# Ahead by a Hair: Preterm Delivery and Maternal Mercury Intake

**Published:** 2007-01

**Authors:** John Tibbetts

Pregnant women often receive confusing information about whether or not they should consume fish and fish oils. Protein and unsaturated fatty acids in fish confer health benefits. Yet numerous studies have suggested that fish consumption is a major source of mercury exposure, and scientists have raised concerns that mercury levels safe for adults could pose a hazard to the developing fetus. Now a new study suggests another possible hazard associated with mercury exposure during pregnancy: preterm delivery **[*EHP* 115:42–47; Xue et al.]**.

The Pregnancy Outcomes and Community Health study, conducted by researchers at Michigan State University, is the first large, community-based study to examine the risk of preterm birth in relation to mercury concentrations among women with low to moderate exposure to the contaminant. This study is also the largest in the United States to correlate fish consumption and maternal hair mercury. Hair levels of total mercury reflect a longer window of contaminant exposure (for example, across the first half of pregnancy) than blood levels, which reflect recent exposure.

The researchers examined maternal hair mercury levels during mid-pregnancy (between weeks 15 and 27) in 1,024 women from 52 prenatal clinics in five Michigan communities. This state borders on four of the five Great Lakes, giving the women easy access to sport-caught fish, which can be relatively high in mercury. Each community included urban, suburban, and rural areas.

The women reported the amount and category of fish they consumed from the beginning of the current pregnancy through the time of the interview. Canned fish was the most frequently eaten fish category during the first six months of pregnancy, followed by fish bought at a store or restaurant. Only 9.2% of women reported consuming sport-caught fish during the first six months of pregnancy, and almost none ate shellfish. These women’s levels of reported fish consumption would be considered moderate to low when compared to populations that subsist on fish.

The researchers took hair samples from close to the scalp to approximate exposure during the pregnancy to date, then assessed them for total mercury levels. They found that total fish consumption correlated positively with mercury levels in hair. Women who delivered before 35 weeks had three times the odds of having hair mercury levels at or above the 90th percentile (0.55–2.5 μg/g), compared with women delivering at term (37 weeks or later). Although the study sample was large, the small number of women delivering before 35 weeks (44) means more studies are needed to test this association.

## Figures and Tables

**Figure:**
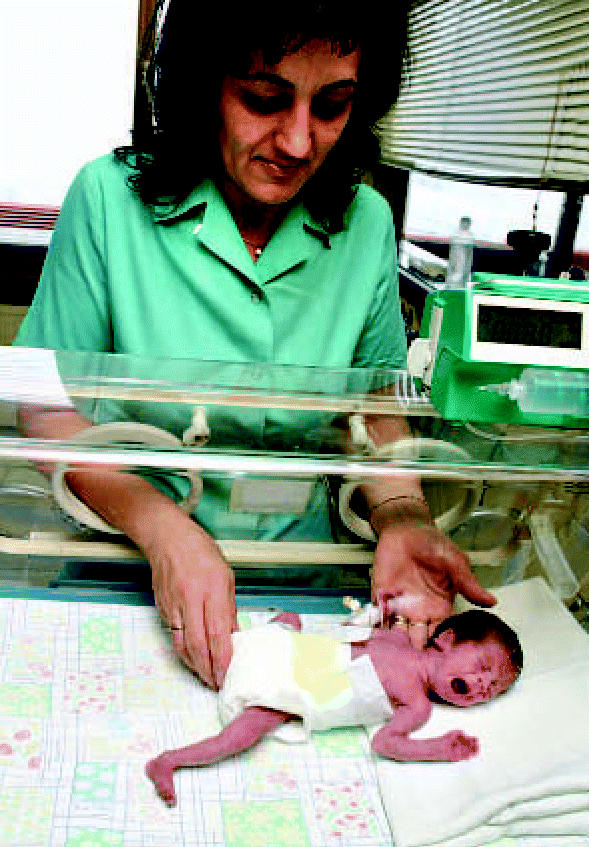
Predictor of preemies? Measures of mercury in maternal hair may predict risk for preterm delivery.

